# Effects of Different Maceration Times on the Chemical and Sensory Characteristics of Cabernet Sauvignon Grapes Wine

**DOI:** 10.3390/foods15081416

**Published:** 2026-04-17

**Authors:** Xiang Chu, Ai Zhang, Yuan Su, Xiangyu Sun

**Affiliations:** College of Enology, Northwest A&F University, Xianyang 712100, China

**Keywords:** Cabernet Sauvignon wine, crushing degree, maceration time, phenolic compounds, aroma compounds

## Abstract

Polyphenols and aroma compounds are major contributors to wine quality and are primarily derived from grape skins and seeds. This study investigated the effects of crushing degree and maceration time on the phenolic and aroma profiles of Cabernet Sauvignon wine. Wines were produced under different crushing degrees (50–100%) and maceration times (5–13 d), and their phenolic and aroma compounds were analyzed by ultraviolet–visible spectrophotometry (UV–Vis) and gas chromatography–mass spectrometry (GC-MS). The results showed that both crushing degree and maceration time significantly affected the extraction and accumulation of these key compounds. A crushing degree of 70–80% combined with 7–9 d of maceration was more suitable for producing wines with a balanced color, aroma, and taste profile. In contrast, complete crushing (100%) and 11 d of maceration were more favorable for enhancing antioxidant potential, with flavanol and total phenol contents reaching 346.6 and 115.9 mg/L, respectively. These findings provide a theoretical basis for optimizing vinification conditions and improving wine quality to meet diverse consumer preferences.

## 1. Introduction

Wine is one of the oldest and most widely consumed traditional alcoholic beverages. Among red wine grape cultivars, Cabernet Sauvignon is one of the most extensively cultivated worldwide because of its thick skin and high phenolic content. The quality of Cabernet Sauvignon wine is primarily evaluated by two intuitive and critical attributes, namely color and aroma. Phenolic compounds are central to the quality formation of Cabernet Sauvignon wine. These compounds, which originate from different grape tissues, can be broadly classified into flavonoids, such as anthocyanins and tannins, and non-flavonoids, such as hydroxycinnamic acids and resveratrol. Phenolic compounds contribute significantly to the color, mouthfeel, flavor complexity, aging potential, and health-related properties of wine. Anthocyanins provide the fundamental color of red wine and contribute to color stabilization through interactions with other phenolic compounds. Tannins determine the structural framework of wine and influence astringency and mouthfeel. In addition, phenolic compounds and their transformation products formed during aging further enrich wine flavor, while a relatively high phenolic content is generally associated with enhanced aging potential. Some phenolic compounds also exhibit potential health-promoting properties, such as antioxidant activity [1]. Therefore, phenolic compounds not only determine the color, astringency, and antioxidant capacity of wine, but also profoundly influence aroma perception through their interactions with volatile compounds [2,3]. Maceration time and crushing degree are two key processing factors affecting these quality attributes. Crushing uses mechanical force to disrupt the cell walls of grape skins and seeds, thereby promoting the release of intracellular substances, including phenolics, sugars, and organic acids, into the must [4]. Wang et al. reported significant differences in the phenolic contents of wines produced from different grape varieties grown in the same region, as well as from the same variety grown in different regions [5]. Zhang et al. showed that the phenolic content of wine is influenced by berry size, the skin-to-seed ratio, and the concentration and composition of phenolic compounds in the pulp, skins, and seeds [6]. They further noted that Cabernet Sauvignon is characterized by relatively high phenolic content, with grape seeds representing the richest source of phenolic compounds. Maceration time regulates the kinetics of solid–liquid mass transfer, whereas crushing degree directly determines the extent of cell wall disruption [7,8]. Both factors can enhance the extraction of phenolic compounds into wine, and their interaction may result in different extraction patterns and compositional changes [9]. Previous studies have investigated the effects of different crushing degrees on phenolic and aroma compounds in Cabernet Sauvignon wine under a fixed maceration time of 5 days. However, systematic research on the combined effects of different crushing degrees and maceration times on phenolic and aroma compounds in wine remains limited.

Color and aroma, the two core indicators of wine quality, have long been recognized through sensory evaluation. However, their formation mechanisms under the regulation of individual processing parameters remain incompletely understood and are still subject to considerable debate. Previous studies have shown that a high crushing degree (>80%) can accelerate the release of phenolic compounds [10]. It has also been reported that extending maceration time beyond 10 days increases total phenolic content, but significantly decreases the retention of key aroma compounds such as β-damascenone. In contrast, Kostecka-Gugala et al. found that the content of phenolic compounds no longer increased significantly when maceration exceeded 4 days [11]. These inconsistent findings raise an important question as to whether prolonged maceration is always beneficial, or whether a moderate maceration duration is sufficient to achieve desirable wine quality. Using response surface methodology, one study demonstrated a significant interaction between crushing degree and maceration time (*p* < 0.01), although this conclusion was limited to the Syrah grape variety. That study did not take into account varietal differences in cell wall structure, and most previous aroma metabolomics analyses have focused primarily on static maceration conditions [12]. At present, relatively few studies have systematically examined the combined effects of crushing degree and maceration time on phenolic and aroma compounds in Cabernet Sauvignon wine, particularly with the aim of identifying an optimal maceration duration and an appropriate crushing degree.

Therefore, this study investigated the effects of different crushing degree gradients (50–100%) and maceration times (5–13 days) on the phenolic and aroma profiles of Cabernet Sauvignon wine. Phenolic compounds were quantified using ultraviolet–visible spectrophotometry (UV–Vis), while aroma compounds were characterized by gas chromatography–mass spectrometry (GC–MS). This controlled experimental design enabled an in-depth evaluation of the extraction patterns of phenolic and aroma compounds in Cabernet Sauvignon wine and provides a scientific basis for optimizing winemaking practices.

## 2. Materials and Methods

### 2.1. Experimental Materials

The experimental material was *Vitis vinifera* L. cv. Cabernet Sauvignon, collected from Row 8 (Code A8) at the Caoxinzhuang Teaching Base of the College of Enology, Northwest A&F University, Yangling, China (34°18′9″ N, 108°5′12″ E) [13]. The vineyard is located in a loess soil region at an altitude of 435–563 m, with a mild climate, sufficient sunlight, a large diurnal temperature range, and a long frost-free period. The annual average temperature is 12.9–13.5 °C, and the annual rainfall is 580–660 mm, of which approximately 60% occurs from July to September [13]. At harvest, the grape berries had a reducing sugar content of 160.36 g/L (expressed as glucose), total acidity of 5.75 g/L (expressed as tartaric acid), a sugar-to-acid ratio of 27.89, and soluble solids content of 16.93%.

### 2.2. Experimental Instruments and Reagents

#### 2.2.1. Experimental Instruments

Startorius BT25S 1/100,000 balance; Wine Color Analyzer (W100), Jinan Haineng Instrument Co., Ltd., Jinan, China; multi-function microplate detector (SpectraMax iD5), Molecular Devices, LLC, San Jose, CA, USA; Anton Paar FTIR Automatic Analyzer (LYZA-5000), Anton Paar GmbH, Graz, Styria, Austria; High-Speed Refrigerated Benchtop Centrifuge (SORVAIL RC-5C-PLUS), Kendro Corporation, New Town, CT, USA; Electric Thermostatic Water Bath (HH.W21.600S), Shanghai Yuejin Medical Instrument Factory, Shanghai, China; Solid Phase Microextraction (SPME) Device, equipped with 57330-U joint handle and DVB/CAR/PDMS extraction fiber (50/30 μm film thickness, 2 cm stableflex), Supelco, Bellefonte, PA, USA; Gas Chromatography-Mass Spectrometry (GC-MS) System, consisting of Agilent 6890 GC and Agilent 5975 MS, Agilent Technologies, Santa Clara, CA, USA; Capillary Column: HP-INNO wax (60 m × 0.25 mm × 0.25 μm), J&W Scientific, Folsom, CA, USA; Blast Drying Oven (DGX-9243BC), Shanghai Nanrong Experimental Equipment Co., Ltd., Shanghai, China; Numerical Control Ultrasonic Cleaner (KH-500DE), Kunshan Hechuang Instrument Equipment Co., Ltd., Kunshan, China.

#### 2.2.2. Experimental Reagents

Reagent: Folinol-Ciocalteu, Solebao Science & Technology Co., Ltd., Beijing, China; Analytical grade reagents including anhydrous copper sulfate, potassium sodium tartrate, sodium hydroxide, glucose, potassium hydrogen phthalate, phenolphthalein, methylene blue, hydrochloric acid, sulfurous acid, and anhydrous sodium carbonate, Beijing Chemical Reagent Co., Ltd., Beijing, China; Standards including gallic acid, catechin, and methylcellulose, Shanghai Yuanye Biotechnology Co., Ltd., Shanghai, China; Treatment reagents including chitosan, Tween-20, and glacial acetic acid; chromatographic grade reagents including methanol and dichloromethane; PVPP, D-gluconolactone; and all aroma standards, Sigma-Aldrich Corporation, St. Louis, MO, USA; Pectinase (LALLZYME EX, activity about 18000 U/g), Lallemand Inc., Montreal, QC, Canada; Active Dry Yeast (CEC01), Angel Yeast Co., Ltd., Yichang, China.

### 2.3. Experimental Method

To ensure sample randomness and representativeness, Cabernet Sauvignon grapevines were selected from six vineyard rows, with six vines chosen from each row. The selected vines in each row were labeled according to a zigzag sampling pattern, starting from the second vine and proceeding at regular intervals. During sampling, ten grape clusters were randomly collected from each labeled vine. From each cluster, grapes were taken from the upper, middle, lower, inner, and outer positions. In total, 100 berries were collected for each sampling to ensure representative sampling [13].

**Figure 1:**
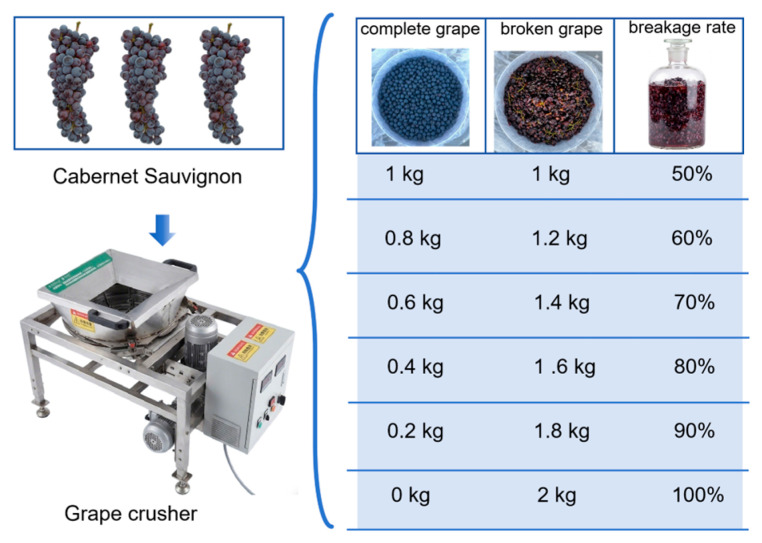
Fermentation methods for grapes with different degrees of fragmentation experimental design.

Mature Cabernet Sauvignon grapes were harvested and vinified on a small scale. After manual destemming, the berries were crushed with a grape crusher. The crushed material was divided into six portions, and intact berries were added back at different proportions to obtain musts with crushing degrees of 50%, 60%, 70%, 80%, 90%, and 100% (Figure 1). SO_2_ was added to the must at 60 mg/L, followed by the addition of pectinase (40 mg/L) after 30 min of sulfitation. The pectinase was pre-activated with grape juice at a 1:1 mass ratio. After another 30 min, the must was subjected to cold maceration at 5 °C for 48 h and then allowed to warm naturally to room temperature. Alcoholic fermentation was initiated by inoculation with active dry yeast at 200 mg/L after activation at 37 °C for 15–30 min. Fermentation was carried out at 16–18 °C. Temperature and specific gravity were monitored every 8 h during fermentation. Skin and pomace separation were performed according to the designed maceration times. Fermentation was terminated by adding SO_2_ at 30 mg/L when the specific gravity dropped below 0.994. The detailed vinification procedure is shown in Table 1.

#### 2.3.1. Determination of Basic Physicochemical

The contents of total sugar, total acidity, volatile acidity, pH, dry extract, and alcohol were determined according to the Chinese national standard GB/T 15038-2006, Methods for Analysis of Wine and Fruit Wine [14]. For each measurement, a 15 mL sample was directly injected into the ATR (Attenuated Total Reflectance) cell using a syringe without prior filtration or dilution.

#### 2.3.2. Determination of Total Phenols in Wine

The total phenolic content was determined using the Folin–Ciocalteu method, and the results were expressed as gallic acid equivalents (GAE, mg/L). The standard calibration curve was described by the regression equation *y* = 0.0087*x* + 0.0001, *R*^2^ = 0.9980. The total phenolic content was calculated as: $\frac{C\times Vdil}{Wsample\times 1000}$.

#### 2.3.3. Determination of Total Anthocyanins in Wine

The total anthocyanin content in the wine samples was determined by the pH differential method, and the results were expressed as cyanidin-3-glucoside equivalents (mg/L). Total anthocyanins content (mg/L) is calculated as the equation of $\frac{A\times Mw\times Vdil\times DF\times 1000}{\in \times l\times m}$. Here, *A* is the difference between the absorbance values at pH 1.0 and pH 4.5, measured at 520 nm and 700 nm, respectively.

#### 2.3.4. Determination of Total Tannins in Wine

The methylcellulose precipitation method was used to measure the content of total tannins in wine, which were expressed as catechin equivalents (mg/L). The standard curve equation was *y* = 0.003*x* − 0.002, *R*^2^ = 0.9943. Total tannin content was calculated as the equation of $\frac{A+0.002}{0.003}$ (mg/L). Here, *A* is the difference in absorbance at 280 nm between the sample before precipitation and the supernatant of the sample after precipitation. A 0.04% methylcellulose solution was used for the measurement.

#### 2.3.5. Determination of Total Flavonoids in Wine

The total flavonoid content in wine was calculated in rutin (mg/L). The measured standard curve equation is *y* = 0.001*x* − 0.0012, *R*^2^ = 0.9957. Total flavonoid content is calculated as the equation of $\frac{A\times DF\times 1000}{\in \times l\times m}(mg/L)$. Here, *A* is the absorbance value of the sample measured at 510 nm.

#### 2.3.6. Determination of Total Flavan-3-ols in Wine

The hydrochloric acid–methanol method was expressed as catechin equivalents (mg/L). The standard curve equation obtained was *y* = 0.0033*x* + 0.0026, *R*^2^ = 0.9993. Total flavan-3-ols content is calculated as the equation of $\frac{A+0.0026}{0.0033}(mg/L)$. Here, *A* is the absorbance value of the sample at 500 nm.

#### 2.3.7. Measurement of Wine Color

The L*, a*, b*, chroma Cab, hue hab, and color difference ΔE*ab of the samples were determined using a W100 wine color analyzer, enabling quantitative analysis and characterization of the wine color.

#### 2.3.8. Determination of Wine Aroma

Volatile compounds were extracted using headspace solid-phase microextraction (HS-SPME) and analyzed by gas chromatography–mass spectrometry (GC–MS). Briefly, 5 mL of wine sample was placed in a vial containing 1 g of NaCl, 10 μL of 4-methyl-2-pentanol as the internal standard, and a magnetic stir bar. After sealing, the vial was equilibrated at 40 °C with stirring at 500 r/min for 10 min. The SPME fiber was then exposed to the headspace above the sample for 30 min for volatile adsorption, followed by thermal desorption in the GC injector for analysis. Qualitative identification was performed using GC-MS software 5.0 in combination with the NIST05 mass spectral library and retention times. Quantitative analysis was conducted based on calibration curves established with authentic standards [15]. The GC oven temperature program was as follows: initial temperature 50 °C, held for 1 min; increased to 220 °C at 3 °C/min; and held for 5 min. The MS conditions were as follows: ion source temperature, 230 °C; ionization mode, electron impact (EI); electron energy, 70 eV; quadrupole temperature, 150 °C; and transfer line temperature, 230 °C.

### 2.4. Statistical Analysis

Statistical analysis was performed using SPSS version 20.0, with Duncan’s multiple range test applied to determine significant differences at a significance level of *p* < 0.05. Graphical representations of the data were generated using Origin 2024 software.

## 3. Result and Discussion

### 3.1. The Impact of Fragmentation on Wine

#### 3.1.1. Basic Physical and Chemical Indicators

The basic physicochemical properties of the experimental Cabernet Sauvignon dry red wines are presented in Table 2. All measured parameters complied with the requirements of the Chinese national standard GB/T 15037-2006 for wine, including volatile acidity ≤ 1.2 g/L (expressed as acetic acid), dry extract ≥ 18 g/L, and residual sugar ≤ 4.0 g/L. As shown in Table 2, the physicochemical properties of Cabernet Sauvignon wines varied among treatments with different crushing degrees [14]. The alcohol content ranged from 10.88% to 11.51% (*v*/*v*), with the highest value observed at the 100% crushing degree and the lowest at the 60% crushing degree. Notably, the residual sugar content was highest in the 60% crushing treatment among all groups, although the underlying reason for this result remains unclear.

Acidity plays an important role in inhibiting microbial growth and maintaining the storage stability of wine. In general, the titratable acidity of red wine should be higher than 4.5 g/L, and all wines produced under different crushing degrees satisfied this requirement. Among the treatments, the wine produced at a crushing degree of 100% exhibited the highest titratable acidity, the lowest volatile acidity, and the lowest pH value [16]. In contrast, the wine produced at a crushing degree of 90% showed the lowest titratable acidity, relatively higher volatile acidity, and the highest pH value. This phenomenon may be related to the enhanced release of minerals and pectic substances from grape skins, seeds, and stems under a high crushing degree. The released minerals may interact with organic acids, while pectic substances may contribute to buffering effects, thereby reducing titratable acidity. Meanwhile, the increased exposure of the juice to oxygen, together with the release of additional nutrients, may favor the growth and oxidative metabolism of acetic acid bacteria, resulting in an increase in volatile acidity. The combined effects of reduced titratable acidity, partial neutralization of free hydrogen ions by minerals, and changes in the buffering system may ultimately lead to an increase in pH [17,18].

Dry extract in wine refers to the total amount of non-volatile solids remaining after the removal of water, ethanol, and other volatile components. It is an important physicochemical parameter for evaluating the level of non-volatile constituents in wine and directly influences its taste, body, and overall quality characteristics. As shown in Table 2, the dry extract content of wines produced under different crushing degrees ranged from 31.07 to 31.97 g/L, with the highest value observed at the 80% crushing degree and the lowest at the 60% crushing degree. This result may be attributed to the fact that a crushing degree of 80% achieved a better balance between the extraction of soluble substances and the retention of desirable components, thereby avoiding the reduction in dry extract associated with either insufficient or excessive crushing [19,20].

#### 3.1.2. Phenolic Content at Different Degrees of Crushing

**Figure 2:**
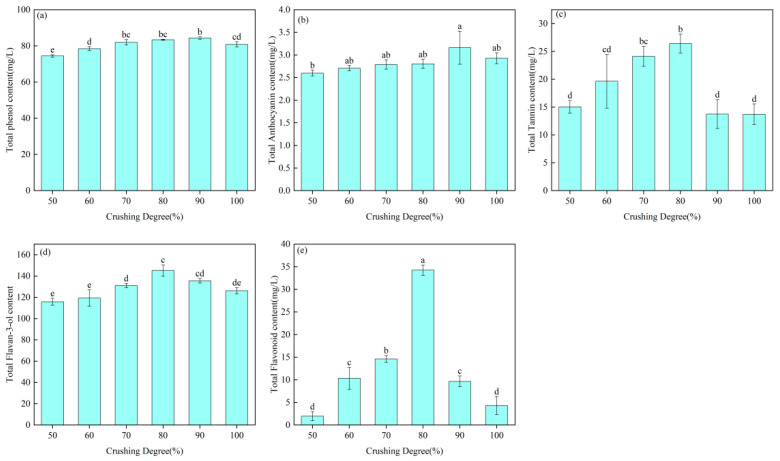
Phenolic composition and content of wines with different degrees of crushing soaked for 5 d. (**a**) Total Phenol; (**b**) Total Anthocyanin; (**c**) Total Tannin; (**d**) Total Flavan-3-ol; (**e**) Total Flavonoid. Values within the same column followed by different lowercase letters are significantly different at *p* < 0.05 using LSD multiple comparison.

Phenolic compounds are a group of natural substances characterized by one or more hydroxyl groups attached to aromatic rings. They are widely found in fruits and vegetables, including grapes, and thus occur abundantly in grape must and wine [21]. As important quality-related constituents, phenolic compounds strongly influence the appearance, color, astringency, bitterness, flavor, and stability of wine [22,23,24]. The phenolic contents of wines obtained under different crushing degrees were analyzed, and the results are shown in Figure 2.

Total phenols are an important class of secondary metabolites with antioxidant, antimicrobial, and preservative properties, and they are also associated with reduced enzyme activity and enhanced disease resistance. As shown in Figure 2, under a fixed maceration time, crushing degree had a significant effect on the total phenol content of the wine. The total phenol content was lowest at 74.40 mg/L at a crushing degree of 50% and reached a maximum of 84.33 mg/L at 90% crushing. From 50% to 90% crushing, the total phenol content showed an increasing trend, indicating that mechanical crushing promoted the extraction of phenolic compounds by disrupting the cellular structure of grape skins. However, when the crushing degree increased further from 90% to 100%, the total phenol content decreased, which may be attributed to the combined effects of oxidation, polymerization, adsorption, and precipitation of the extracted phenolic compounds [18].

Anthocyanins are the pigments responsible for the color of red wine. During maceration and fermentation, anthocyanins located mainly in grape skins are progressively released into the wine matrix as the ethanol concentration increases [25]. As shown in Figure 2b, the anthocyanin content reached a maximum of 3.16 mg/L at a crushing degree of 90%. When the crushing degree increased from 50% to 90%, the anthocyanin content in the wine showed a corresponding upward trend. This result is consistent with previous studies, indicating that an increased crushing degree can promote anthocyanin extraction into wine [26,27]. However, when the crushing degree further increased from 90% to 100%, the anthocyanin content declined. This decrease may be attributed to the enhanced extraction of seed-derived tannins at higher crushing intensities during prolonged maceration. These tannins may interact with anthocyanins to form more stable tannin–anthocyanin (T–A) complexes, thereby reducing the measurable anthocyanin content [28].

Tannins are water-soluble phenolic compounds produced through plant secondary metabolism and are mainly distributed in grape skins, seeds, and stems. Figure 2c shows the tannin contents of wines obtained under different crushing degrees. The tannin content reached its maximum at a crushing degree of 80% and its minimum at 100%. The increasing trend observed from 50% to 80% crushing may be attributed to the enhanced mechanical disruption of grape skins and seeds at higher crushing intensities, which damaged the cell wall structure and promoted the release of condensed tannins from these tissues. However, the tannin content decreased when the crushing degree increased from 80% to 100% and became relatively stable between 90% and 100%. This phenomenon may be associated with the formation of stable tannin–anthocyanin (T–A) complexes, which reduced the amount of free tannins detected in the wine [28].

Flavan-3-ols are an important class of flavonoids biosynthesized through the flavonoid metabolic pathway. They not only act as copigments that help prevent color deterioration and improve the color stability of wine, but also exhibit antioxidant activity and potential health-promoting effects [25,29]. As shown in Figure 2d, the variation pattern of flavan-3-ol content was similar to that of tannins. The flavan-3-ol content increased as the crushing degree rose from 50% to 80%, which may be attributed to the greater mechanical disruption of grape skins and seeds at higher crushing intensities. This disruption likely damaged the cell wall structure and promoted the release of flavan-3-ols from these tissues. However, when the crushing degree further increased from 80% to 100%, the flavan-3-ol content declined. This reduction may be associated with a series of reactions, including oxidation, polymerization with anthocyanins, reactions with aldehydes, precipitation with tartrates, and adsorption onto yeast cell walls, ultimately leading to the formation of insoluble complexes [30].

Flavonoids are derivatives of the pyran ring, and the diversity of their substituents gives rise to a wide range of flavonoid compounds. In addition to their biological functions, such as antioxidant, antimicrobial, and UV-protective activities, flavonoids also play an important role in the sensory quality of wine [31]. As shown in Figure 2e, the total flavonoid content increased rapidly as the crushing degree rose from 50% to 80%, but decreased markedly when the crushing degree further increased from 80% to 100%. Previous studies have shown that fermentation following heat maceration and prolonged maceration can increase the total flavonoid content in wine, whereas repeated stirring and high-temperature fermentation may lead to its reduction [32]. Accordingly, it can be inferred that the increase in flavonoid content from 50% to 80% crushing was associated with enhanced extraction resulting from greater tissue disruption. In contrast, the decline observed from 80% to 100% crushing may be attributed to oxidative degradation caused by increased exposure of reaction substrates to oxygen or by changes in the activity of related enzyme systems [33]. The biosynthesis and metabolic regulation of grape flavonoids are influenced by multiple factors, including light, temperature, water availability, hormones, postharvest storage conditions, and the transcriptional regulation of related metabolic pathways, all of which have been studied to some extent. However, relatively few studies have focused on the effects of vinification techniques on flavonoid content in wine. Therefore, the underlying mechanism responsible for the present phenomenon remains unclear and warrants further investigation.

#### 3.1.3. Color Changes Under Different Degrees of Fragmentation

The color parameters of the wine samples were determined using a W100 wine color analyzer, and the results are presented in Table 3. Lightness (L*) reflects the brightness of the wine color, with values closer to 0 indicating lower lightness and values closer to 100 indicating higher lightness. Under different crushing degree treatments, all wine samples exhibited relatively high L* values, indicating good gloss, although significant differences were observed among the groups.

The red–green coordinate (a*) represents the red–green component of color, where values closer to −100 indicate a stronger green hue and values closer to 100 indicate a stronger red hue. The a* values of wines produced under different crushing degrees ranged from 11.86 to 13.59, indicating a strong red component in all samples, with significant differences among treatments. The lowest a* value was observed at a crushing degree of 50%, whereas the highest was found at 100%. The yellow–blue coordinate (b*) represents the yellow–blue component, with values closer to −100 indicating a stronger blue hue and values closer to 100 indicating a stronger yellow hue. The b* values ranged from 5.59 to 7.29, suggesting that the wines also exhibited a relatively pronounced yellow component. Significant differences were detected among treatments, with the lowest b* value at 100% crushing and the highest at 80% crushing.

Chroma (C*ab), which is determined by both a* and b*, reflects the color saturation or intensity of the sample in the color space. A higher C*ab value indicates greater color saturation. The results showed that the wines had relatively low chroma overall, although significant differences were observed among the groups. The lowest chroma was found at 60% crushing degree, whereas the highest was recorded at 80%.

Hue angle (h*ab) is a key attribute of color that describes the overall color tendency. In red wine, h*ab generally ranges from 0° to 90°. Lower h*ab values indicate that the color is closer to purplish red or ruby red, which are typical of young red wines, whereas higher values indicate a shift toward tile red or brick red, which are commonly associated with aged wines. As shown in Table 3, the hue values of the samples ranged from 0.39° to 0.52°, indicating a fresh ruby-red appearance. Significant differences were observed among treatments, with the lowest hue value at 100% crushing degree and the highest at 60%.

The total color difference (ΔE*ab) is a comprehensive parameter derived from L*, a*, and b*, and is used to quantify the overall color difference between samples in the CIELAB color space according to the following equation: ΔE*ab = √[(ΔL*)^2^ + (Δa*)^2^ + (Δb*)^2^]. A larger ΔE*ab value indicates a greater visual color difference. According to color difference theory, when ΔE*ab exceeds 6, the difference can generally be clearly perceived by the human eye. In the present study, although significant differences in ΔE*ab were observed among treatments, all values were below 6. This indicates that the visual color differences among the wine samples were not obvious.

#### 3.1.4. Qualitative and Quantitative Analysis of Aromatic at Different Degrees of Fragmentation

**Figure 3:**
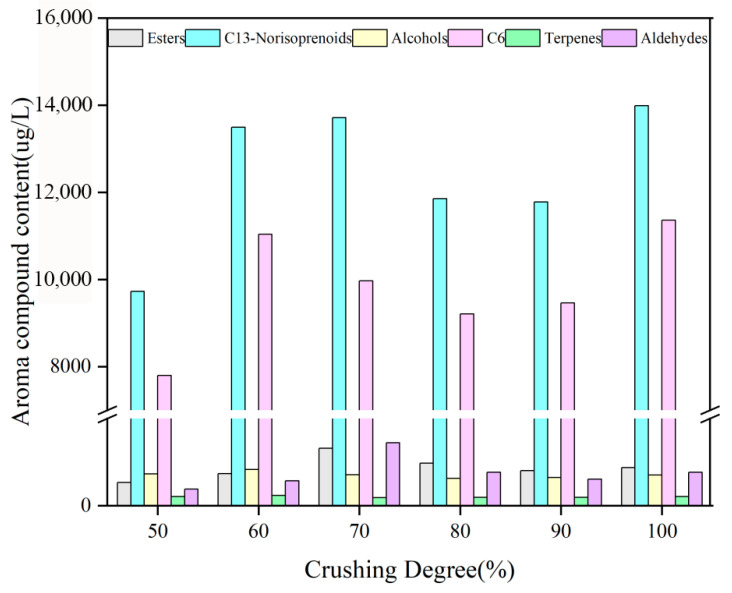
Aromatic components and content of wine after 5 days of maceration with different degrees of crushing.

As shown in Figure 3, grape crushing degree had a significant regulatory effect on the composition of volatile aroma compounds in Cabernet Sauvignon wine, and different chemical classes exhibited distinct response patterns. In this study, a total of 46 volatile compounds were identified and classified into six groups: C6 compounds (4 compounds), alcohols (8 compounds), esters (10 compounds), aldehydes (9 compounds), terpenes (11 compounds), and norisoprenoids (4 compounds). The total aroma content varied markedly with crushing degree, reaching the highest level at 100% crushing (13,987.62 μg/L, group a), which was 43.8% higher than that at 50% crushing (9725.73 μg/L, group f). Notably, the total aroma contents at 60% (13,491.69 μg/L, group c) and 70% crushing (13,717.56 μg/L, group b) were also close to the maximum, whereas those at 80% (11,850.98 μg/L, group d) and 90% crushing (11,781.08 μg/L, group e) decreased by approximately 14%. This nonlinear trend may be explained by two opposing effects. On the one hand, complete tissue disruption at 100% crushing likely promoted the release of intracellular constituents, such as lipids and glycosidically bound aroma precursors, thereby enhancing the formation of C6 compounds and esters. On the other hand, relatively high crushing degrees (70–90%) may have accelerated oxidative reactions, such as the conversion of aldehydes into corresponding acids, or inhibited yeast metabolic activity, thereby reducing ester biosynthesis efficiency, since esters are mainly formed during alcoholic fermentation [34]. Although the total aroma content was highest at 100% crushing, the distribution of key sensory compounds, particularly esters, aldehydes, and terpenes, suggests that crushing degrees of 60–70% may be more favorable for aroma quality. Therefore, aroma quality should be evaluated not only on the basis of total concentration, but also by considering odor activity values (OAVs) and the interactions among aroma compounds.

Grape crushing degree also significantly affected the formation of C6 compounds in Cabernet Sauvignon wine. The total content of C6 compounds showed a bimodal distribution, with peaks at 60% crushing (11,038.66 μg/L) and 100% crushing (11,360.12 μg/L). Among them, 1-hexanol was the major contributor to the green, grassy aroma, with an OAV of 2.12. Although the total C6 concentration was highest at 100% crushing, the maximum levels of (Z)-2-hexen-1-ol, (Z)-3-hexen-1-ol, and hexanal were observed at 70%, 60%, and 70% crushing, respectively. These results indicate that a moderate crushing degree may be more effective in optimizing aroma complexity and balance than complete crushing.

Most alcohols in wine are by-products of yeast fermentation, and within an appropriate concentration range, they make a positive contribution to wine aroma [35]. In the present study, the total alcohol content reached its maximum at a crushing degree of 60% (832.80 μg/L), mainly due to the substantial contribution of 1-octen-3-ol (OAV = 15.03). Among the detected alcohols, 1-octanol, which is associated with citrus and rose notes, 1-nonanol, which contributes rose and orange aromas, and phenylethyl alcohol, which imparts rose and pollen-like notes, all reached their highest concentrations at 60% crushing. These compounds contributed markedly to the total alcohol content.

Esters are an important class of volatile compounds in wine and play a key role in enhancing aroma complexity [36]. In this study, the total ester content reached its maximum at a moderate crushing degree of 70% (1315.15 μg/L), mainly driven by the combined contributions of 3-methyl-1-butyl acetate (OAV = 2.43) and ethyl hexanoate (OAV = 12.55). Except for methyl octanoate, butyl acrylate, and methyl benzoate, which showed no significant differences among treatments, almost all other detected esters reached their highest concentrations at 70% crushing. The nine aldehydes detected in this study showed a response pattern similar to that of the esters.

Among the identified terpene compounds, only α-terpineol, citronellol, geraniol, and p-mentha-1,8-dien-7-ol showed significant differences under different crushing degree treatments, whereas the remaining compounds did not vary significantly. The peak concentrations of these four compounds, as well as the total terpene content, were all observed at 60% crushing. Among the four C13-norisoprenoids detected, only β-macrocarpone and 1-octen-3-one showed significant differences, and both reached their maximum concentrations at moderate crushing degrees (60–70%). The total C13-norisoprenoid content also peaked at 70% crushing.

In conclusion, the qualitative and quantitative analyses indicated that a moderate crushing degree (60–70%) significantly promoted the accumulation of floral- and fruity-aroma-related compounds in Cabernet Sauvignon wine. Terpenes, such as geraniol, and esters, such as ethyl hexanoate, reached their highest concentrations at 60–70% crushing, with OAVs of 190.38 and 12.55, respectively, indicating their dominant contributions to floral and fruity aroma characteristics. C13-norisoprenoids, represented by β-macrocarpone (OAV = 1234), also showed the highest aroma activity at 70% crushing, further enhancing the complexity of roasted nut and sweet apple notes. In contrast, although complete crushing (100%) produced the highest total aroma content (13,987.62 μg/L), the excessive accumulation of C6 compounds, particularly 1-hexanol (OAV = 2.12), may intensify green and grassy notes and thereby mask more delicate aroma attributes, ultimately reducing overall aroma quality. Previous studies have likewise shown that grape crushing degree strongly influences the extraction of terpenes and C13-norisoprenoids. Moderate crushing is more conducive to preserving terpene integrity, whereas excessive crushing may accelerate oxidative degradation and weaken floral expression. These findings further confirm the important regulatory role of crushing intensity in the release and transformation of key aroma precursors.

During Cabernet Sauvignon vinification, grape crushing degree significantly regulated the formation and accumulation of volatile aroma compounds, thereby influencing the sensory characteristics of the wine. Moderate crushing (60–70%) achieved a balanced disruption of grape tissues while effectively promoting the accumulation of esters and terpenes, whose relative proportions reached 38% and 12%, respectively, representing the highest levels among the treatment groups. This suggests that an appropriate level of mechanical disruption favors yeast metabolism for ester formation while preserving part of the grape-derived terpene fraction. In contrast, severe crushing markedly increased the proportion of C_6_ aldehydes to 18%. As most C_6_ aldehydes are oxidative cleavage products of fatty acids and are commonly associated with undesirable green and grassy notes, this result indicates that excessive crushing may impair aroma quality. Meanwhile, the terpene proportion under severe crushing decreased to 8%, suggesting that excessive tissue disruption may accelerate the loss or oxidative degradation of volatile terpenes. By contrast, mild crushing (<60%) resulted in insufficient release of intracellular constituents and a relatively low total aroma content, making it difficult to form a complex and well-balanced aroma profile. Previous studies have shown that crushing intensity not only affects the extraction of phenolic compounds, but also indirectly alters the transformation pathways of aroma precursors by modulating the oxidation level. In particular, activation of polyphenol oxidase may aggravate the accumulation of oxidative products such as C_6_ aldehydes, which is consistent with the aroma characteristics observed under severe crushing in this study. Therefore, precise control of grape crushing degree is essential for balancing oxidation risk and aroma expression.

According to the experimental results, different crushing degrees caused different levels of damage to grape skins and seeds, resulting in significant differences in the contents of total phenols, tannins, total flavonoids, flavan-3-ols, and anthocyanins in the wine. The contents of flavan-3-ols, tannins, and total flavonoids reached their maximum values at 80% crushing, whereas total anthocyanins and total phenols peaked at 90% crushing. In terms of color parameters, the a* value reached its maximum at 100% crushing and its minimum value of 11.86 at 50%, while the b* value was lowest at 100% crushing and highest at 80%. Chroma (C*ab) was lowest at 60% crushing and highest at 80%, whereas hue angle (h*ab) was lowest at 100% crushing and highest at 60%. However, the overall color difference among wines was limited, with ΔE*ab values below 6, indicating that the visual differences among samples were not readily distinguishable. Aroma analysis further showed that esters, aldehydes, and terpenes were the major contributors to the volatile profile, and these key aroma groups generally reached their highest levels at crushing degrees of 60–70%.

### 3.2. The Effect of Soaking Time on Wine

#### 3.2.1. Basic Physicochemical Indicators

The basic physicochemical properties of the experimental Cabernet Sauvignon dry red wine samples are presented in Table 4. All measured parameters met the requirements of the Chinese national standard GB/T 15037-2006 for wine, including volatile acidity ≤ 1.2 g/L (expressed as acetic acid), dry extract ≥ 18 g/L, and residual sugar ≤ 4.0 g/L [14].

As shown in Table 4, the basic physicochemical properties of Cabernet Sauvignon wines varied with maceration time. The alcohol content ranged from 11.50% to 12.14% (*v*/*v*). Within the maceration period of 5–11 days, alcohol content increased with prolonged maceration time, reaching its maximum at 11 days and its minimum at 5 days. Residual sugar showed a fluctuating trend, decreasing initially, then increasing, and finally decreasing again as maceration time was extended. This pattern may be related to differences in the rate of sugar conversion by yeast under different maceration durations [37].

The major acids in wine include tartaric acid, malic acid, lactic acid, and citric acid. As the backbone of wine taste, these acids contribute to freshness, enhance aroma expression, and shape overall wine style. As shown in Table 4, titratable acidity gradually decreased with prolonged maceration time, from 4.91 g/L at 5 days to 4.47 g/L at 13 days. This reduction may be attributed to the interaction of titratable acids, such as tartaric and malic acids, with potassium ions during maceration, leading to the formation of precipitates and, consequently, a decrease in titratable acidity [30]. These results indicate that wine acidity can be modulated to some extent by adjusting maceration time. Volatile acidity (expressed as acetic acid) showed a trend of first decreasing and then increasing, reaching its highest value (0.53 g/L) at 5 days and its lowest value (0.38 g/L) at 9 days. In contrast, pH remained relatively stable across treatments, ranging from 3.82 to 3.85, indicating that maceration time had only a limited effect on wine pH. This is consistent with previous findings showing that pH and residual sugar are not significantly influenced by maceration time in Cabernet Sauvignon wine [38].

Dry extract is an important parameter reflecting the body and fullness of wine. As shown in Table 4, the dry extract content first increased and then decreased with maceration time, reaching its maximum value (32.43 g/L) at 7 days and its minimum value (31.60 g/L) at 5 days. This pattern may be associated with the increased extraction of phenolic and other non-volatile compounds during the early stage of maceration. With further extension of maceration time, some high-molecular-weight phenolic substances may undergo polymerization or oxidation, resulting in reduced solubility and precipitation, and thus a gradual decrease in dry extract content [39].

#### 3.2.2. Changes in Phenolic Content at Different Soaking Times

**Figure 4:**
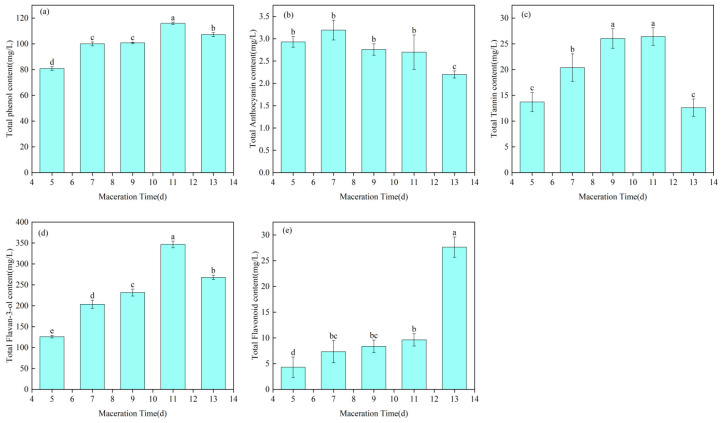
Phenolic composition and content of wines with 100% crushing degree at different maceration times. (**a**) Total Phenol; (**b**) Total Anthocyanin; (**c**) Total Tannin; (**d**) Total Flavan-3-ol; (**e**) Total Flavonoid. Values within the same column followed by different lowercase letters are significantly different at *p* < 0.05 using LSD multiple comparison.

Phenolic compounds comprise a broad class of substances, including tannins, lignin, phenylpropanoids and their polymers, and flavonoids. In wine, these compounds contribute to astringency and structural complexity, enhance wine stability, and may also exert health-promoting effects, such as antioxidant and anti-inflammatory activities. In the present study, maceration was conducted for 5, 7, 9, 11, and 13 days under otherwise identical conditions. The changes in five groups of phenolic compounds in the experimental Cabernet Sauvignon dry red wines are shown in Figure 4a–e, namely total phenols, total anthocyanins, total tannins, total flavan-3-ols, and total flavonoids.

As shown in Figure 4a, the total phenol content first increased and then decreased with prolonged maceration time, which is consistent with the results reported by Fan et al. [40]. The total phenol content reached its maximum at 11 days and then declined slightly at 13 days. This trend may be explained by the progressive extraction of phenolic compounds during maceration. In the early stage, soluble monomeric phenolic acids and flavonoids in grape skins are readily extracted. As maceration proceeds and pectinase continues to act, more bound phenolics or those entrapped within the cell wall matrix are gradually released into the wine, resulting in an increase in total phenol content. However, at longer maceration times, phenolic compounds may undergo precipitation or re-adsorption onto grape skins. In addition, some soluble polyphenols may be adsorbed by cell wall components, oxidized and polymerized, or precipitated at an early stage, leading to a slight decline in total phenol content. These results indicate that extending maceration can promote total phenol extraction, whereas excessively prolonged maceration may instead reduce the final content [41].

As shown in Figure 4b, the total anthocyanin content exhibited an overall downward trend with increasing maceration time from 7 to 13 days. This may be attributed to the high water solubility of anthocyanins, which enables their rapid release during the early stage of maceration. During the middle and later stages, anthocyanins may copolymerize with tannins to form more stable polymeric pigments or copigmentation complexes, thereby reducing the measured content of free anthocyanins [42]. In addition, anthocyanins may degrade under the influence of dissolved oxygen, metal ions, and enzymes, or be adsorbed by grape cell walls and lees, resulting in a decrease in measurable total anthocyanin content [43]. Previous studies on different grape varieties have similarly shown that maceration promotes pigment release at the early stage, whereas prolonged maceration does not necessarily increase the free anthocyanin content and may even lead to a decline after the peak value is reached [44].

As shown in Figure 4c, the tannin content gradually increased from 5 to 11 days with prolonged maceration time. During maceration, the system was initially dominated by the aqueous phase, and tannins from grape skins were progressively released into the wine. Therefore, a longer maceration period allowed more extensive diffusion and enhanced tannin extraction. However, the tannin content reached its maximum at 11 days and then decreased at 13 days. This decline may be attributed to prolonged exposure to oxygen, which promoted tannin oxidation and the formation of precipitates through polymerization or solvent saturation. Yiyang et al. reported high absorbance of stable pigments after 6 days of maceration in intensively crushed Marquette grapes subjected to enzymatic treatment [45]. This observation is generally consistent with the high tannin accumulation observed in the present study [46].

As shown in Figure 4d, the total flavan-3-ol content exhibited an overall increasing trend, reaching a maximum at 11 days and then decreasing slightly at 13 days. The increase from 9 to 11 days was more pronounced than that from 5 to 7 days, which may be attributed to the gradual breakdown of seed coats and the increase in ethanol concentration with prolonged maceration, both of which facilitate the extraction of flavan-3-ols [47]. Flavan-3-ols, including catechin and epicatechin, are important contributors to bitterness, astringency, and antioxidant activity, and also serve as the structural subunits of condensed tannins [48]. Compared with anthocyanins, their extraction is generally slower and requires longer skin and seed contact time. In addition, low temperature and SO_2_ can inhibit oxidation and help preserve monomeric flavan-3-ols, while pectinase promotes cell wall degradation and facilitates their release. With further extension of maceration time, some monomeric flavan-3-ols may undergo oxidative condensation or become adsorbed onto lees, resulting in a decline in the measured concentration of soluble monomers relative to the peak level [49]. A previous study on the cold maceration kinetics of Cabernet Sauvignon similarly reported that extending cold maceration increased catechin and epicatechin levels during fermentation and in the finished wine, although this increase was partly offset by adsorption and polymerization at longer maceration times [38].

As shown in Figure 4e, the total flavonoid content exhibited an overall increasing trend with prolonged maceration time. This may be attributed to the activation of enzymes such as β-glucosidase during maceration, which can hydrolyze bound flavonoid glycosides into free flavonoids, thereby increasing their solubility and facilitating their release into the wine matrix. In addition, prolonged maceration promoted cell wall disruption, leading to the release of more flavonoids [50]. Another possible explanation is that extended maceration increased the contact area between the solvent and intracellular components, shortened the diffusion distance, and accelerated the transfer of flavonoids from the interior of the cells into the surrounding medium. Some studies have suggested that extending maceration has limited or no significant effect on flavonol content. However, when the analysis is expanded to total flavonoids, the combined effects of cell wall degradation, fermentation, temperature, and increasing ethanol concentration over time may promote the release of a broader range of flavonoid compounds [38]. Overall, these results indicate that appropriate control of maceration time can effectively enhance the extraction and accumulation of phenolic compounds in wine.

#### 3.2.3. Color Changes Under Different Soaking Time

As shown in Table 5, the wine samples under different maceration time treatments all exhibited relatively high lightness, indicating good brightness, although significant differences were observed among the groups. The a* value represents the red component of color and is commonly used to evaluate chromatic changes in red wine, whereas the b* value mainly reflects the yellow component.

As shown in Table 5, the a* values of wines produced under different maceration times ranged from 11.92 to 14.77, indicating a strong red component in all samples. Overall, the a* value showed a decreasing trend with prolonged maceration time, although it increased initially from 5 to 7 days and then declined from 7 to 13 days. The initial increase may be attributed to the progressive extraction of pigments, such as anthocyanins and flavonoids, from grape skins into the must as maceration proceeded. The subsequent decline may be associated with pigment saturation and the polymerization of anthocyanins with tannins, leading to the formation of insoluble precipitates and, consequently, a reduction in redness. This trend is consistent with previous findings reported by Chen et al. [48]. The b* values ranged from 4.25 to 5.59, indicating a measurable yellow component in the wine color, with significant differences among treatments. The b* value was lowest at 9 days of maceration and highest at 5 days. The chroma (C*ab) of the wines was relatively low overall and generally decreased with prolonged maceration time, although significant differences were observed among the groups. The lowest chroma was found at 7 days, whereas the highest was observed at 13 days. The hue angle (h*ab) ranged from 0.29° to 0.39°, indicating that the wines maintained a fresh ruby-red appearance. Significant differences were observed among treatments, with the lowest hue at 9 days of maceration and the highest at 5 days. The total color difference (ΔE*ab) ranged from 2.94 to 3.71. Although significant differences were detected among treatments, all ΔE*ab values were below 6, indicating that the visual color differences among the wine samples were not obvious.

#### 3.2.4. Qualitative and Quantitative Analysis of Aromatic with Different Soaking Times

In this study, the aroma profiles of Cabernet Sauvignon dry red wines produced with different maceration times (5, 7, 9, 11, and 13 days) were analyzed by GC–MS. A total of 52 volatile compounds were identified and classified into six groups according to their chemical structures and aroma characteristics: C6 compounds (4), alcohols (8), esters (10), aldehydes (9), terpenes (11), and C13-norisoprenoids (4). Both the total concentration and composition of these volatile compounds varied significantly with maceration time, indicating that maceration time plays an important regulatory role in wine aroma formation.

**Figure 5:**
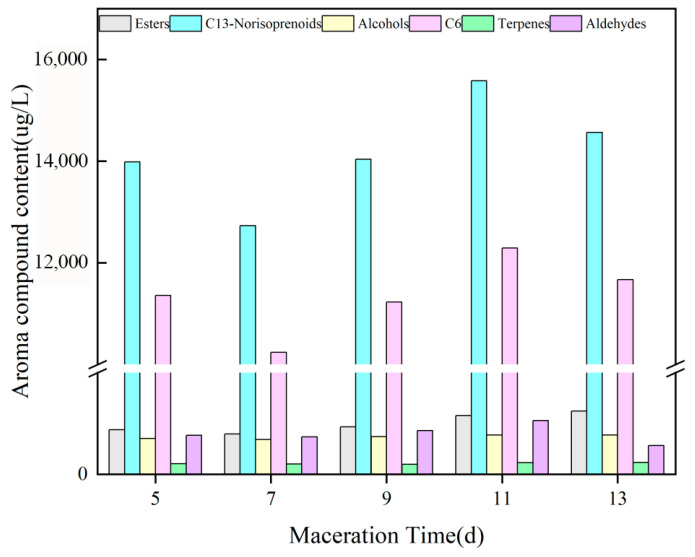
Aromatic components and content of wine with 100% crushing at different maceration time.

As shown in Figure 5, the total volatile content showed a trend of first decreasing, then increasing, and finally decreasing again with prolonged maceration time, reaching a maximum at 11 days (15,582.06 μg/L), which was approximately 22.4% higher than the minimum value observed at 7 days (12,729.66 μg/L). Extending maceration time may enhance the release of aroma precursors from grape tissues and promote yeast secondary metabolism, thereby increasing the accumulation of volatile compounds. However, excessively long maceration, such as 13 days, may lead to the loss of some active aroma compounds through oxidation or ester hydrolysis, resulting in a decline in total volatile content. This pattern is consistent with previous findings in grape varieties such as Merlot and Cabernet Franc, in which the richest aroma profile was obtained at moderate maceration durations [51].

C6 compounds played a major role in the volatile profile of the wines. For example, trans-2-hexenal is formed from linolenic acid through the action of lipoxygenase-related enzymes, including hydroperoxide lyase, and has a high odor activity value, thereby exerting a strong influence on wine aroma [52]. These compounds are generally associated with green, grassy, and vegetal notes [53]. In the present study, C6 compounds accounted for a large proportion of the total volatile compounds and already reached a relatively high level (11,360.12 μg/L) at the early maceration stage (5 days). Their content then gradually decreased, increased again to a maximum at 11 days, and declined after 13 days, which is consistent with the results reported by Wang et al. [54]. This trend may be explained by the rapid release of membrane lipids from grape skins and seeds during the early stage of cold maceration, followed by their conversion into C6 aldehydes and alcohols through the lipoxygenase pathway [55]. As fermentation progressed, yeast-mediated reduction of aldehydes and their further conversion through condensation or other reactions likely contributed to a decrease in the final concentration of C6 compounds [56]. Although C6 compounds constituted a major fraction of the volatile profile, they are often considered less desirable from a sensory perspective because excessive levels may mask fruity and floral notes. In addition, the total aldehyde content reached its maximum value (1057.26 μg/L) at 11 days of maceration, whereas terpene compounds peaked at 13 days.

Alcohols were abundant in all samples, reaching their highest concentration at 11 days of maceration (771.16 μg/L), with no significant difference compared with that at 13 days (773.19 μg/L). This suggests that alcohol accumulation no longer increased markedly after 11 days of maceration. Alcohols are mainly formed as by-products of fermentation through amino acid and sugar metabolic pathways, and at appropriate concentrations they can enhance wine aroma complexity [57]. Among the detected alcohols, phenylethyl alcohol, which imparts rose-like notes, and 1-octen-3-ol, which contributes mushroom-like aroma, were the most characteristic compounds. In particular, the odor activity value of 1-octen-3-ol exceeded 10, indicating a significant contribution to the overall aroma profile through mushroom and earthy notes. Phenylethyl alcohol increased markedly during the middle and late stages of maceration (9–11 days), suggesting that a moderate maceration duration is beneficial for the accumulation of floral aroma compounds. Previous studies have similarly shown that appropriate extension of maceration can increase the availability of aroma precursors for yeast metabolism, thereby promoting alcohol formation [58]. Tao et al. also reported that esters made the greatest contribution to the aroma profile of Chardonnay wine [59].

In this study, different maceration times applied to Cabernet Sauvignon grapes resulted in significant differences in total phenols, tannins, total flavonoids, flavan-3-ols, anthocyanins, and other quality-related parameters in the resulting wines. The contents of total flavan-3-ols, tannins, total anthocyanins, and total phenols all showed an initial increase followed by a decline. Among these, total flavan-3-ols, total phenols, and tannins reached their maximum values at 11 days of maceration, whereas total anthocyanins peaked at 7 days. In contrast, total flavonoids exhibited a continuous increasing trend throughout the maceration period. These results indicate that the extraction and accumulation of phenolic compounds in Cabernet Sauvignon wine were most pronounced at a maceration time of 11 days. According to the statistical analysis of wine color parameters, the a* and C*ab values generally decreased with prolonged maceration time, indicating progressive changes in chromatic characteristics during vinification. Specifically, the a* value increased initially, reached its maximum at 7 days, and then declined with further extension of maceration time. Aroma analysis showed that esters, aldehydes, and terpenes were the major contributors to the volatile profile of the wines, and the total aroma content remained relatively high at longer maceration times, particularly at 11–13 days.

## 4. Conclusions

This study evaluated the effects of crushing degree (50–100%) and maceration time (5–13 d) on the phenolic and aroma profiles of Cabernet Sauvignon wine by UV–Vis spectrophotometry and GC-MS. Both factors significantly affected phenolic extraction, volatile composition, and color-related parameters. Under different crushing degree treatments, most phenolic compounds showed an initial increase followed by a decline. Total anthocyanins reached the highest level at 90% crushing, whereas total flavan-3-ols, tannins, and total flavonoids peaked at 80% crushing. Total phenols reached their maximum at 90% crushing. In parallel, aroma composition was strongly dependent on crushing degree, with esters and aldehydes reaching the highest levels at 70% crushing, while excessive crushing reduced the accumulation of higher alcohols and terpenes. Overall, a crushing degree of 70–80% was the most favorable for achieving a balanced phenolic and aroma profile. At a fixed crushing degree of 100%, maceration time also markedly influenced wine composition. Total anthocyanins peaked at 7 d, tannins at 9 d, and total flavan-3-ols and total phenols at 11 d, whereas total flavonoids reached the highest level at 13 d. The total volatile content was also highest at 11 d, indicating that moderate maceration favored the accumulation of both phenolic and aroma compounds, whereas excessive maceration may impair aroma quality. Taken together, these results suggest that vinification conditions should be adjusted according to the target wine style. A crushing degree of 70–80% combined with 7–9 d of maceration is recommended for wines with a balanced color, aroma, and taste profile, whereas 100% crushing combined with 11 d of maceration is more suitable for maximizing antioxidant-related phenolics.

## Figures and Tables

**Table 1:** Test wine numbering.

Effect of Different Crushing Degrees on Wine	Number	T1	T2	T3	T4	T5	T6
	Crushing Degree/%	50	60	70	80	90	100
	Maceration Time/days	5	5	5	5	5	5
Effect of Different Maceration Times on Wine	Number	T6	T7	T8	T9	T10	
	Crushing Degree/%	100	100	100	100	100	
	Maceration Time/days	5	7	9	11	13	

**Table 2:** Physicochemical characteristics of *Cabernet Sauvignon* wine with different degrees of crushing and a maceration time of 5 d.

Crushing Degree	Alcoholic Degree/%vol	Residual Sugar Content/(g·L^−1^)	Titratable Acidity Content/(g·L^−1^)	Volatile Acidity/(g·L^−1^) (Calculated as Acetic Acid)	pH	Dry Extract Content/(g·L^−1^)
50	11.20 ± 0.05 bc	0.53 ± 0.06 ab	4.74 ± 0.05 b	0.57 ± 0.05 ab	3.88 ± 0.02 b	31.33 ± 0.06 ab
60	10.88 ± 0.05 e	0.70 ± 0.20 a	4.58 ± 0.03 c	0.60 ± 0.04 ab	3.91 ± 0.01 a	31.07 ± 0.50 b
70	11.25 ± 0.05 bc	0.50 ± 0.17 ab	4.72 ± 0.01 b	0.61 ± 0.03 a	3.91 ± 0.01 a	31.67 ± 0.42 ab
80	11.15 ± 0.05 cd	0.60 ± 0.10 ab	4.53 ± 0.04 cd	0.57 ± 0.02 ab	3.92 ± 0.01 a	31.97 ± 0.25 a
90	11.10 ± 0.05 d	0.43 ± 0.06 b	4.51 ± 0.01 d	0.61 ± 0.08 ab	3.92 ± 0.01 a	31.70 ± 0.36 ab
100	11.51 ± 0.03 a	0.53 ± 0.12 ab	4.91 ± 0.01 a	0.53 ± 0.03 b	3.82 ± 0.02 c	31.60 ± 0.35 ab

Values within the same column followed by different lowercase letters are significantly different at *p* < 0.05 using LSD multiple comparison.

**Table 3:** CIELAB parameters of Cabernet Sauvignon wine with different crushing levels after 5 days of soaking.

Crushing Degree/%	L*	a*	b*	C*_ab_	h*_ab_/(°)	ΔE*_ab_
50	87.49 ± 0.45 ab	11.86 ± 0.14 d	5.70 ± 0.21 c	13.16 ± 0.18 e	0.45 ± 0.02 c	2.10 ± 0.33 c
60	87.42 ± 1.12 ab	12.34 ± 0.06 c	7.00 ± 0.37 ab	14.19 ± 0.15 c	0.52 ± 0.03 a	3.21 ± 0.16 b
70	88.20 ± 0.48 a	12.32 ± 0.08 c	5.95 ± 0.14 c	13.69 ± 0.12 d	0.45 ± 0.01 c	2.95 ± 0.30 b
80	86.33 ± 1.47 b	13.59 ± 0.07 a	7.29 ± 0.45 a	15.43 ± 0.23 a	0.49 ± 0.03 ab	3.82 ± 0.40 a
90	87.44 ± 0.47 ab	13.03 ± 0.10 b	6.55 ± 0.16 b	14.58 ± 0.14 b	0.47 ± 0.01 bc	3.12 ± 0.09 b
100	86.70 ± 1.43 ab	13.56 ± 0.46 a	5.59 ± 0.42 c	14.67 ± 0.25 b	0.39 ± 0.04 d	3.02 ± 0.27 b

Values within the same column followed by different lowercase letters are significantly different at *p* < 0.05 using LSD multiple comparison.

**Table 4:** Basic physicochemical indicators of 100% crushed Cabernet Sauvignon wine at different maceration time.

Maceration Time/d	Alcoholic Degree/%vol	Residual Sugar Content/(g·L^−1^)	Titratable Acidity Content/(g·L^−1^)	Volatile Acidity/(g·L^−1^) (Calculated as Acetic Acid)	pH	Dry Extract Content/(g·L^−1^)
5	11.51 ± 0.03 b	0.53 ± 0.12 a	4.91 ± 0.01 a	0.53 ± 0.03 a	3.82 ± 0.02 b	31.60 ± 0.35 b
7	11.78 ± 0.02 c	0.47 ± 0.06 a	4.58 ± 0.04 b	0.43 ± 0.04 b	3.85 ± 0.01 a	32.43 ± 0.32 a
9	11.89 ± 0.06 c	0.57 ± 0.06 a	4.53 ± 0.04 bc	0.38 ± 0.00 b	3.83 ± 0.01 ab	32.23 ± 0.40 ab
11	12.14 ± 0.08 a	0.63 ± 0.25 a	4.50 ± 0.03 cd	0.41 ± 0.05 b	3.84 ± 0.01 a	31.93 ± 0.40 ab
13	12.03 ± 0.09 b	0.50 ± 0.00 a	4.47 ± 0.01 d	0.44 ± 0.08 b	3.84 ± 0.01 ab	31.90 ± 0.14 ab

Values within the same column followed by different lowercase letters are significantly different at *p* < 0.05 using LSD multiple comparison.

**Table 5:** CIELAB parameters of Cabernet Sauvignon wine at 100% crushing at different maceration time.

Maceration Time/d	L*	a*	b*	C*_ab_	h*_ab_/(°)	ΔE*_ab_
5	86.70 ± 1.43 b	13.56 ± 0.46 b	5.59 ± 0.42 a	14.67 ± 0.25 b	0.39 ± 0.04 a	3.02 ± 0.27 c
7	86.53 ± 0.38 b	14.77 ± 0.11 a	4.67 ± 0.11 b	15.49 ± 0.14 a	0.31 ± 0.01 c	3.71 ± 0.04 a
9	88.02 ± 0.24 a	13.16 ± 0.08 c	3.94 ± 0.07 d	13.74 ± 0.08 c	0.29 ± 0.01 c	3.08 ± 0.15 c
11	88.33 ± 0.20 a	12.66 ± 0.08 d	4.41 ± 0.04 bc	13.40 ± 0.08 d	0.34 ± 0.01 b	2.94 ± 0.14 c
13	89.09 ± 0.23 a	11.92 ± 0.04 e	4.25 ± 0.08 c	12.66 ± 0.06 e	0.34 ± 0.01 b	3.38 ± 0.22 b

Values within the same column followed by different lowercase letters are significantly different at *p* < 0.05 using LSD multiple comparison.

## Data Availability

The original contributions presented in this study are included in the article. Further inquiries can be directed to the corresponding authors.
